# Comparing literature-guided, LLM-assisted, and data-driven models for predicting angler pressure

**DOI:** 10.1371/journal.pone.0357450

**Published:** 2026-10-09

**Authors:** Azar Taheri Tayebi, Julia S. Schmid, Sean Simmons, Mark S. Poesch, Mark A. Lewis, Pouria Ramazi

**Affiliations:** 1 Department of Mathematics and Statistics, Brock University, St. Catharines, Ontario, Canada; 2 Department of Mathematical and Statistical Sciences, University of Alberta, Edmonton, Alberta, Canada; 3 Angler’s Atlas, Goldstream Publishing, Prince George, British Columbia, Canada; 4 Department of Renewable Resources, University of Alberta, Edmonton, Alberta, Canada; 5 Departments of Mathematics and Statistics and Biology, University of Victoria, Victoria, British Columbia, Canada; 6 Department of Mathematics and Statistics and Department of Biological Sciences, University of Calgary, Calgary, Alberta, Canada; Texas A&M University, UNITED STATES OF AMERICA

## Abstract

This study compared three models–a literature-guided, a large language model (LLM)-assisted, and a data-driven model–all developed using Bayesian networks as the framework for predicting angler pressure measured by the number of boats observed in aerial surveys. The models used meteorological data from 98 lakes in Ontario, Canada, during 2018 and 2019, as well as platform-derived variables, including angler-reported fishing trips, fishing duration, and catch rate, together with lake webpage views. In five-fold cross-validation, no pairwise difference in fold-level accuracy among the three models remained significant after Holm adjustment. These findings suggest that LLM-assisted model construction has potential to support ecological prediction when paired with empirical validation.

## Introduction

Sustainable recreational fisheries management relies on understanding and regulating angler activity [1–3]. A key aspect of this involves managing angler pressure, which refers to the cumulative effects of fishing activities. Recreational boating contributes to angler pressure, as boats often serve as a primary means for anglers to access fishing locations [4]. Increased recreational boating activity can amplify angler pressure, potentially leading to overfishing, disruptions in fish behavior, and environmental degradation through impacts such as anchoring damage, noise pollution, and fuel emissions [5].

Aerial surveys are a popular method to measure boating activities by counting anglers or their boats from airplanes, helicopters, or drones [6]. Nonetheless, aerial surveys can be costly, weather-dependent, limited in detail, and require substantial human and operational resources, including fuel and airplane hire [7,8].

To address these limitations, there is growing interest in harnessing technology-driven approaches to gather complementary data. Citizen-reporting angler smartphone applications (apps) and online platforms enable the collection of low-cost, large-scale, and near-real-time data on angler trips, catch rates, and fishing duration [9–11]. Notably, using the framework of Bayesian networks (BNs) [12], found a direct probabilistic relationship between waterbody webpage views and aerially observed boat counts, and related machine-learning work showed that website visits can predict angler presence [13]; together, these findings suggest that online platform activity may contain information not captured by meteorological variables alone. This is consistent with recreation-demand and angler site-choice frameworks, in which recreation decisions depend on expected benefits, costs, site attributes, and available information [14–17]. However, the potential of webpage views and other citizen-sourced data to accurately predict boating activity and associated angler pressure remains underexplored.

The BN in [12] was fully data-driven and can be used for prediction. However, a model based on peer-reviewed literature, supplemented with intuitive ecological relationships that may not be explicitly covered in the literature, could potentially be more robust as it relies on well-established general principles. The framework of BNs does allow for the incorporation of expert knowledge into the model, by manually constructing the structure of the BN (the connections in the network). The parameters (the conditional distributions of the variables) can still be learned either completely from the data, through informative prior distributions, or by explicitly defining certain relationships between variables [18–21].

Recent advancements in artificial intelligence (AI), particularly in natural language processing, offer another avenue for constructing predictive models. Large language models (LLMs), such as ChatGPT, can process massive amounts of information and synthesize patterns that may escape human analysts or traditional data-driven approaches [22–24]. In principle, LLMs could be engaged similarly to human experts, providing structural insights for a BN through patterns learned from diverse sources, including scientific literature and historical datasets [25].

Despite this progress, no study has systematically compared the predictive performance of literature-guided, LLM-assisted, and data-driven models for recreational fisheries. To address this gap, we developed and evaluated three BN models to predict the number of boats, as a proxy for angler pressure, on 98 lakes in Ontario, Canada, spanning 2018 and 2019. To this end, we formulated the following hypotheses:

- Null Hypothesis (*H*_0_): There is no difference in prediction accuracy among the literature-guided, LLM-assisted, and data-driven models.
- Alternative Hypothesis (*H*_1_): Prediction accuracy varies among the three approaches.

We used citizen-sourced angler activity data from the Angler’s Atlas website and the MyCatch mobile app, combined with meteorological data and aerial survey counts, to train and evaluate the three models.

## Materials and methods

We combined aerial-survey, citizen-sourced, and meteorological data, aggregated the observations by week, and discretized the resulting variables. We evaluated the three BN models using stratified five-fold cross-validation. The literature-guided and LLM-assisted structures were fixed before cross-validation, whereas their parameters and both the structure and parameters of the data-driven BN were estimated within each training partition. We also evaluated a webpage-only BN, linear regression, and random forest (Fig 1). This project, including the use of the dataset, was iewed and approved by the Research Ethics Board of the Alberta Research Information Services (ARISE, University of Alberta), study ID MS5_Pro00102610.

**Fig 1:**
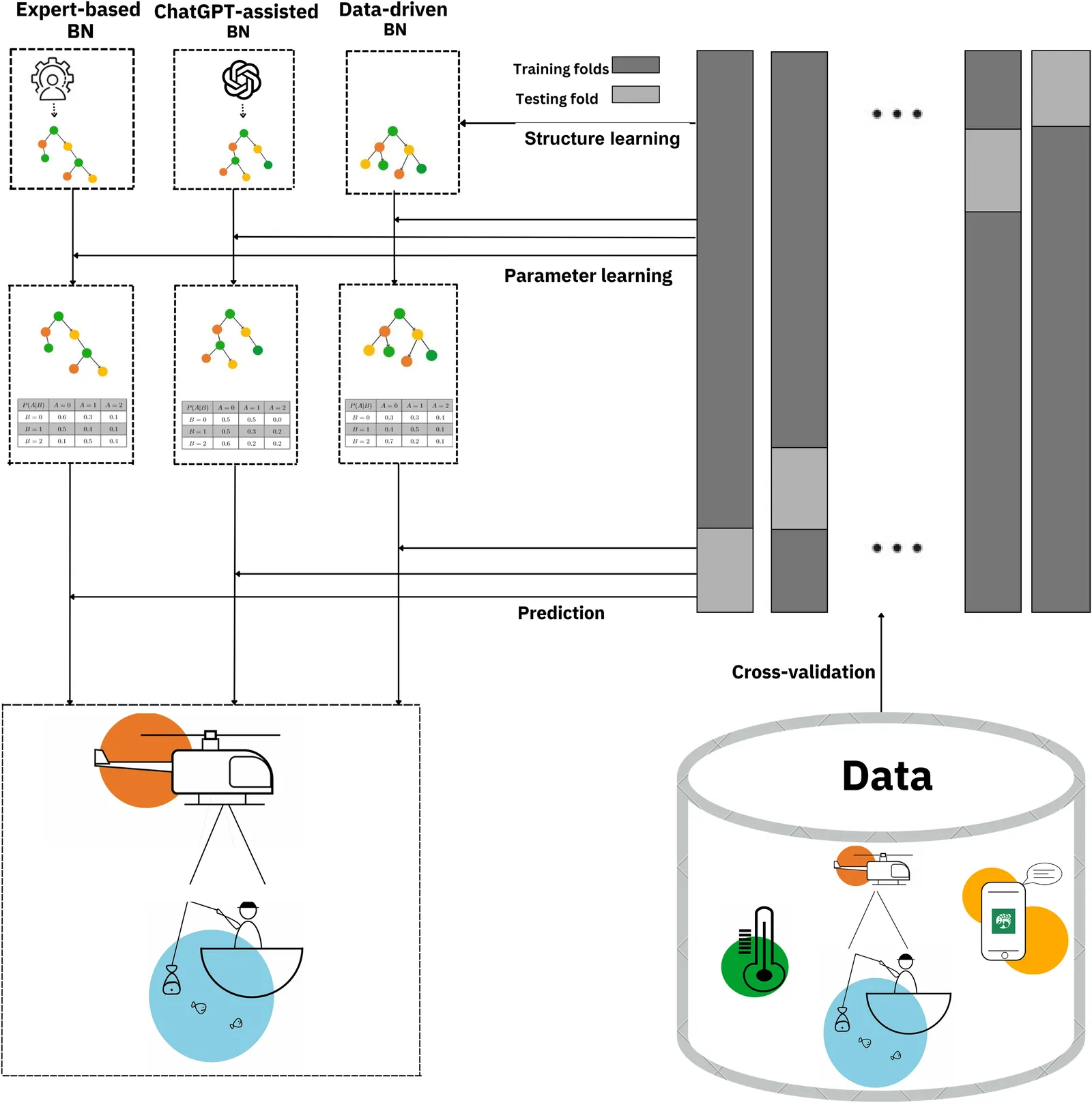
The process of predicting the number of boats reported in aerial surveys using literature-guided (expert-based), LLM-assisted, and data-driven Bayesian networks (BNs). The data, collected from aerial surveys (coral), citizen reports (yellow), and meteorology (green), are divided into training and testing sets for five-fold cross-validation. For each fold, the parameters of the literature-guided and LLM-assisted BNs, as well as the structure and parameters of the data-driven BN, are learned from the training set. The test set is then used to assess the predictive performance of each approach in predicting the target variable.

### Data

We used the dataset described in [12], which is publicly available in [26]. The aerial survey dataset included information from 98 lakes in Ontario, each exceeding 50 hectares in size, sampled as part of the Ontario-wide Broad-scale Monitoring (BsM) program [27] to ensure comprehensive spatial and temporal representation. Lakes were chosen based on a stratified random sampling approach to cover a variety of management zones and capture variability across different lake sizes and periods. Data collection occurred over 78 randomly selected days, covering both weekend-holiday and weekday periods from June 2018 to August 2019 during BsM cycle 3 [4]. During aerial surveys, observed boats were categorized as angling or non-angling recreational craft when classification was possible. The boat-count variable used in this analysis was the count of boats categorized as angling vessels.

In addition, data reported by anglers were sourced from the Angler’s Atlas online platform (www.anglersatlas.com) and the MyCatch mobile app. Established in 1999 by Goldstream Publishing Incorporated in Prince George, British Columbia, Canada, the Angler’s Atlas platform provides extensive information on 330,000 waterbodies across Canada via its website (www.anglersatlas.com). Each waterbody is represented by a dedicated webpage containing details such as its location, commonly targeted species, and markers indicating hotspots and boat launch points. These webpages are publicly accessible without requiring a membership. Anglers can utilize them for trip planning or to share information about their fishing activities. The webpage-views variable was defined as the weekly average of the daily count of unique views of a waterbody’s webpage. Multiple views by the same user on a single day were counted as a single view. For each waterbody-day, this average was calculated over the seven-day window ending on the aerial-survey date; thus, it represents recent pre-survey and same-day online interest rather than only same-day webpage activity. This reflects the typical timeframe anglers use for trip planning [28].

The MyCatch app facilitates real-time data collection by enabling anglers to report trip-specific information such as location, fishing duration, and catch outcomes directly through their smartphones [29]. Anglers could also report trips with no catches. This study considered only data from completed trips, defined as those with fully reported trip information. Reporting a trip for a specific waterbody indicated that the trip took place somewhere within that waterbody. We refer to the collected app- and website-derived data as “citizen-sourced data” for ease of exposition. This term is used only as a shorthand; the app-derived variables and webpage-view variable were retained as separate variables throughout the analysis and were not combined into a single fishing-effort measure.

Anglers reported a total of 8,621 fishing trips spanning 1,670 distinct waterbodies and 310 unique days between May 19, 2018, and August 29, 2019. For each waterbody and day, the dataset included the total number of trips, as well as details for each trip including the number of fish caught and the duration of fishing. Using these inputs, the following metrics were calculated for each day: total fishing duration in hours, total number of fish caught, and the mean catch rate in fish per hour.

Meteorological data were collected as potential predictive variables using the BioSIM tool [30]. BioSIM is software for weather-driven simulation using geographic weather data. The four nearest weather stations within 300 km of each waterbody’s centroid were selected, and the data were adjusted for differences in elevation, latitude, and longitude and calibrated to long-term variability. Historical daily weather observations with bilinear interpolation were used [31]. The variables obtained were those identified in other studies as related to or influencing angler activity, including degree days [32], daily air temperature [33], wind speed [34], precipitation [35], solar radiation [36], and relative humidity [35]. After matching the sources and removing observations without an aerial-survey count, the source dataset contained 1,128 lake-day observations. Because more than half of the observations were zero for several daily variables, all analyses used weekly aggregates. App-derived and aerial-survey counts were summed within lake-week, whereas webpage views and meteorological variables were averaged over days with survey observations. The resulting dataset contained 320 observations and 11 variables (Table 1).

**Table 1:** Variables used to construct the weekly Ontario dataset.

Category	Name	Description	Unit
**Angler Activity**	App catch rate (mean)	Mean citizen-sourced number of fish caught per hour within a lake-week	fish/hour
	App number of trips (total)	Total citizen-sourced trips within a lake-week	trips/ week
	App fishing duration (total)	Total citizen-sourced fishing hours within a lake-week	hours/ week
	Webpage views (mean)	Mean daily number of unique views of the waterbody webpage over the preceding seven days	views/ day
	Number of boats (total)	Total angling boats recorded in aerial surveys within a lake-week	boats/ week
**Auxiliary**	Air temperature (mean)	Mean daily air temperature for the waterbody within the lake-week	°C
	Total precipitation (mean)	Mean daily liquid and solid precipitation within the lake-week	mm
	Wind speed (mean)	Mean daily wind speed within the lake-week	km/hour
	Relative humidity (mean)	Mean daily relative humidity within the lake-week	%
	Solar radiation (mean)	Mean daily solar radiation within the lake-week	W/m^2^
	Degree days (mean)	Cumulative sum, from April 1, of (daily mean temperature – 5 °C) on days when mean temperature exceeds 5 °C	°C

Each non-binary weekly variable was discretized into Low, Medium, and High categories using quantile cut points [37]. For total precipitation, zeros formed the first category and positive values were divided between the other two categories. The weekly boat-count target contained 110 Low, 103 Medium, and 107 High observations. For correlation calculations, the ordered categories were coded as Low = 1, Medium = 2, and High = 3; this coding was used only to quantify monotonic association between webpage-view category and boat-count category. Weekly distributions of five key discretized variables are reported in S2 Table.

### Learning Bayesian networks

A BN is a pair $ℬ=(𝒢,P_{ℬ})$ where

- 𝒢=(𝒱,ℰ) is a directed acyclic graph (BN structure) over the random variables 𝒱 represented by nodes, and ℰ is the set of links representing probabilistic dependence between the connected variables;
- $P_{ℬ}$ is the joint probability distribution over all variables. In practice this joint distribution is stored as one conditional-probability table (CPT) per node, each CPT giving the probability of that node’s states for every combination of its parents’ states [38].

See the Appendix for an example of a BN (S1 Fig and S1 Table).

The process of learning a BN generally involves two steps: first, estimating the structure, known as structure learning, and second, estimating the parameters, known as parameter learning [39,40]. The BN structure can be learned either manually or automatically from data, resulting in a “data-driven” BN. Manual learning involves specifying the network structure based on existing literature and the expert knowledge of practitioners. Automatic learning involves using algorithms to infer and optimize the network structure from datasets [41]. We constructed two manual BN structures, the literature-guided and LLM-assisted BNs, and one data-driven BN structure.

### Train and test

We divided the weekly dataset into five approximately equal, stratified subsets, or “folds,” and conducted five cross-validation experiments [42]. In each experiment, one fold was held out as the test set and the other four together formed the training set. The literature-guided and LLM-assisted BN structures were specified before cross-validation, but their parameters were estimated anew from each training set; both the structure and parameters of the data-driven BN were learned anew from each training set.

For every observation in the held-out fold, the trained BN used the observed predictor vector 𝐱 as evidence and exact inference returned P(Low∣𝐱), P(Medium∣𝐱), and P(High∣𝐱), where 𝐱 denotes the observed predictor values for that observation. The scored class was the posterior mode, $\text{arg}\max_{c}P(c|𝐱)$; exact ties were resolved in the fixed order Low, Medium, High, and no posterior draws were used. The predicted class was then compared with the observed class to calculate accuracy for that fold:


$$\begin{array}{c} Accuracy=\frac{\text{number of correct predictions}}{\text{total number of predictions}}. \end{array}$$

Here, a prediction was correct only when the predicted category exactly matched the observed category.

Within each training partition, the no-information classifier assigned every test observation to the most frequent training class. For each of 5,000 paired-bootstrap replicates, we sampled the 320 held-out cases with replacement, kept the model and baseline predictions paired for each sampled case, and recomputed the difference between their accuracies. The 2.5th and 97.5th percentiles of these differences formed the 95% confidence interval [43]. Letting *b* denote cases correctly classified only by the model and *c* cases correctly classified only by the baseline, the one-sided exact form of McNemar’s test used b∣(b+c)~Binomial(b+c,0.5) [44,45]. The six resulting *p*-values were adjusted with Holm’s step-down procedure [46].

#### Manual construction of a literature-guided BN structure.

Here, “literature-guided” refers to a manually specified BN structure informed by peer-reviewed literature on recreational fisheries, outdoor recreation behaviour, and meteorological influences on angler activity. To construct a literature-guided BN structure, we initiated the process by providing an empty network over the candidate variables. We then conducted a review of the existing peer-reviewed literature to identify the potential relationships among the variables [29,34,35,47–50]. Whenever a relationship between two variables was supported by the literature, a corresponding link was added between them in the network; for example, a supported relationship between webpage views and number of boats was represented as a BN link between those two nodes. Subsequently, a meeting was held to incorporate practical insights and intuitive ecological relationships that might not have been explicitly described in existing literature. These considerations supplemented the BN structure primarily derived from literature.

#### The manual construction of a BN structure based on LLM principles.

For the structure of the LLM-assisted BN, we used ChatGPT version 4 on July 29, 2024. We provided the prompt “Create a Bayesian network containing the following variables in recreational fisheries,” and listed the original candidate variables along with their descriptions. ChatGPT, as it explained, analyzed the relationships between these variables based on domain knowledge, weather patterns, and human behavior influences, then proposed a detailed structure outlining the conditional dependencies among them. The archived response is reproduced in S2 Fig.

#### The automatic construction of a BN structure based on data.

For each training partition, comprising the four training folds together, we used the Silander–Myllymäki algorithm to find the BN structure with the highest Bayesian Information Criterion (BIC) score over that training dataset [51]. We used learn.network in the bnstruct package in R version 4.4.1 [52].

#### Impact of webpage views on aerial survey predictions.

To benchmark the predictive contribution of webpage views, we constructed a two-node BN containing a single link from “webpage views” to “number of boats.” We call this the “webpage-only BN.”

As contextual analogue checks, rather than external validation of the modeled Angler’s Atlas variable, we conducted two supplementary analyses using publicly available visitation, fishing-effort, and Wikipedia pageview data. First, we used monthly National Park Service (NPS) Visitor Use Statistics for 19 U.S. national parks from 2016–2024 and retained records with statistic code TRV, representing monthly recreation visits [53]. Each park-month was paired with monthly Wikimedia pageviews for the corresponding English Wikipedia article page using the Wikimedia Analytics API [54]. The final NPS recreation-analogue dataset contained 2,160 park-month observations. The parks, NPS codes, and Wikipedia article pages are listed in S8 Table.

Second, we used NOAA Marine Recreational Information Program (MRIP) wave-level recreational fishing-effort estimates from 2016–2024 [55,56]. The response variable was ESTRIPS, the estimated number of recreational angler trips. We retained records with STATUS == “FINAL” and positive ESTRIPS, producing 7,078 observations. Each MRIP record corresponded to a combination of year, two-month wave, sub-region, state, fishing mode, and area fished. Fishing modes included charter boat, party boat, private/rental boat, and shore. The MRIP area-fished categories included inland saltwater or brackish waters, ocean areas within or beyond the 3-mile state territorial sea boundary, ocean areas within or beyond the 10-mile boundary used for some jurisdictions, and records with unknown area fished. Monthly Wikimedia pageviews were obtained for five English Wikipedia article pages related to fishing: Fishing, Angling, Recreational_fishing, Fishing_rod, and Fishing_license [54]. Pageviews for these five topics were summed and aggregated to MRIP two-month waves. We also created a one-wave lagged pageview variable.

#### Parameter learning.

Within each training partition, CPTs were estimated using a symmetric Dirichlet prior (bnlearn:: bn.fit, method = “bayes”, equivalent sample size = 5) [57]. Sensitivity analyses used equivalent sample sizes of 1 and 10 and unsmoothed maximum-likelihood estimation (S7 Table).

### Two other machine learning models

To ensure that the predictions of the three main models were not an artifact of the BN framework, we additionally trained linear regression and random forest models within the same five-fold cross-validation [58,59]. The linear regressions were constructed using the lm function and qr decomposition method in R. The random forest models were created using the randomForest function from the randomForest package in R [60]. The random forest model consisted of 500 decision trees.

### Assessing the prediction performance of the models

Accuracy was the principal overall performance measure. Balanced accuracy and macro-F1 for the three primary BNs were summarized in the main text; Tables – in the Appendix report complete balanced accuracy and macro-F1 results, class-specific precision and recall, Cohen’s kappa, Hand–Till multiclass AUC, predicted-class proportions, and pooled out-of-fold confusion matrices [61,62]. We compared fold-level accuracy across the six non-baseline models—the literature-guided, LLM-assisted, data-driven, and webpage-only BNs, linear regression, and random forest—using a Friedman test. Within each fold, the six models were ranked by accuracy; models tied within a fold were assigned the average of the ranks they jointly occupied [63]. A significant Friedman test was followed by paired Wilcoxon tests with Holm adjustment.

## Results

The literature-guided and recorded LLM-assisted weekly structures, together with the completed partially directed acyclic graph (CPDAG) learned from the fold-5 training data as a representative data-driven structure, are shown in Fig 2. Table 2 compares the variable pairs connected by literature, common-sense intuition, the LLM, and data. The webpage-views node was directly connected to number of boats in both fixed structures and in every data-driven fold; the fold-1–4 data-driven CPDAGs are shown in S3 and S4 Figs.

**Table 2:** Variable connections proposed by peer-reviewed literature (L), common-sense intuition (I), the recorded LLM response (C), and data (D). Five data-driven BNs were generated in five-fold cross-validation of the weekly dataset; *D*(*n*) indicates that the corresponding variable pair was connected in *n* of the five learned structures.

Air temperature	–										
App catch rate	C, D(2)	–									
App fishing duration	I, C, D(1)	I, C, D(2)	–								
App number of trips	C	L, I	I, D(5)	–							
Degree days	C	–	–	–	–						
Number of boats	I	C	D(2)	D(4)	–	–					
Relative humidity	C, D(2)	C, D(1)	C	–	D(3)	–	–				
Total precipitation	D(5)	D(2)	L, I	D(2)	–	I, D(3)	L, I, C, D(1)	–			
Webpage views	C, D(4)	D(2)	I, D(2)	C, D(5)	C, D(5)	I, C, D(5)	D(1)	–	–		
Solar radiation	C, D(4)	C	C	–	D(5)	–	D(5)	D(5)	–	–	
Wind speed	D(5)	C, D(2)	L, I, C	–	D(5)	I	C, D(1)	–	–	D(4)	–
	Air temperature	App catch rate	App fishing duration	App number of trips	Degree days	Number of boats	Relative humidity	Total precipitation	Webpage views	Solar radiation	Wind speed

Literature-supported links were based on associations reported for app trips and catch rate [47], precipitation and fishing duration [50], precipitation and relative humidity [48], and wind speed and fishing duration [34]. Direction is not represented in this pairwise table.

**Fig 2:**
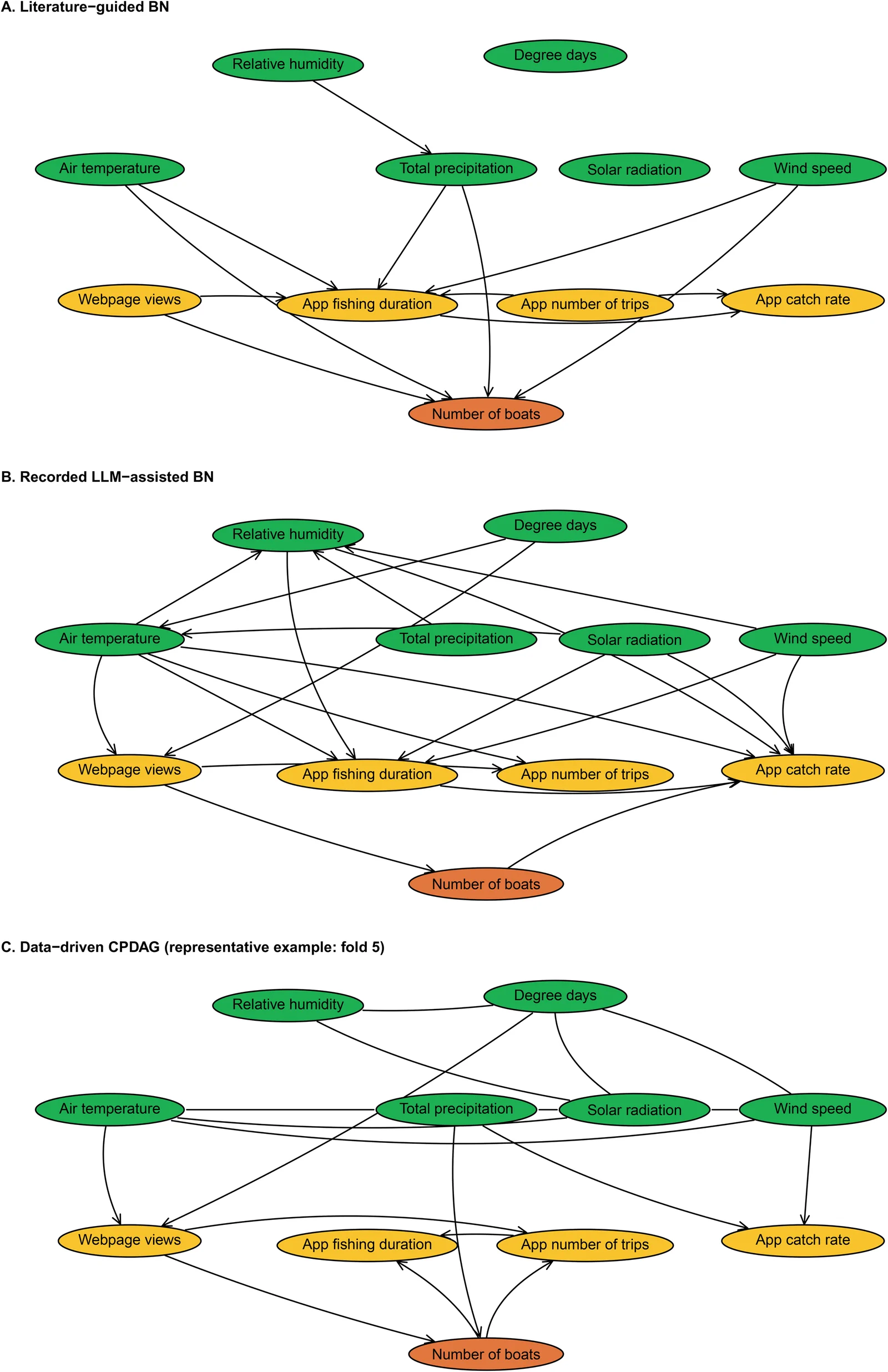
Weekly Bayesian-network structures: (A) the literature-guided BN, (B) the recorded LLM-assisted BN, and (C) the data-driven CPDAG learned from the fold-5 training data, shown as a representative example. Green nodes represent meteorological variables, yellow nodes represent webpage- and app-derived variables, and the coral node represents the aerial-survey boat-count target.

Weekly webpage-view and boat-count categories were positively associated: Pearson’s *r* = 0.563 (95% CI 0.483–0.633, *p* < 0.001) and Spearman’s ρ=0.562 (bootstrap 95% CI 0.479–0.638, *p* < 0.001).

In the supplementary NPS recreation analogue, across 19 national parks, the median Spearman correlation between monthly NPS recreation visits and monthly Wikimedia pageviews was 0.447 for same-month pageviews and 0.439 for one-month lagged pageviews. Same-month correlations were positive for 18 of 19 parks and significant at *p* < 0.05 for 16 parks; one-month lagged correlations were positive for all 19 parks and significant at *p* < 0.05 for 17 parks.

In the supplementary NOAA MRIP fisheries check, the aggregate correlation analysis used 54 two-month waves based on final MRIP ESTRIPS effort estimates and summed Wikimedia pageviews for five fishing-related topics. For effort summarized across all fishing modes, Spearman correlations were 0.106 for same-wave pageviews and 0.166 for one-wave lagged pageviews, with corresponding *p*-values of 0.446 and 0.230. For private/rental boat effort, Spearman correlations were 0.092 for same-wave pageviews and 0.157 for one-wave lagged pageviews, with corresponding *p*-values of 0.506 and 0.257.

The weekly majority-class baseline accuracy was 0.344. Pooled out-of-fold accuracies were 0.534 for the data-driven BN, 0.469 for the literature-guided BN, 0.447 for the LLM-assisted BN, 0.553 for the webpage-only BN, 0.519 for linear regression, and 0.541 for random forest. All six models exceeded the paired fold-specific baseline after Holm adjustment ($p_{adj}\leq 0.0047$). Fold-level mean accuracies (SD) in the same order were 0.534 (0.060), 0.469 (0.085), 0.447 (0.074), 0.554 (0.091), 0.519 (0.043), and 0.541 (0.063) (Fig 3 and S6 Table). The Friedman test detected an overall difference in accuracy ($\chi^{2}(5)=13.49$, *p* = 0.0192), but no pairwise Wilcoxon comparison remained significant after Holm adjustment.

**Fig 3:**
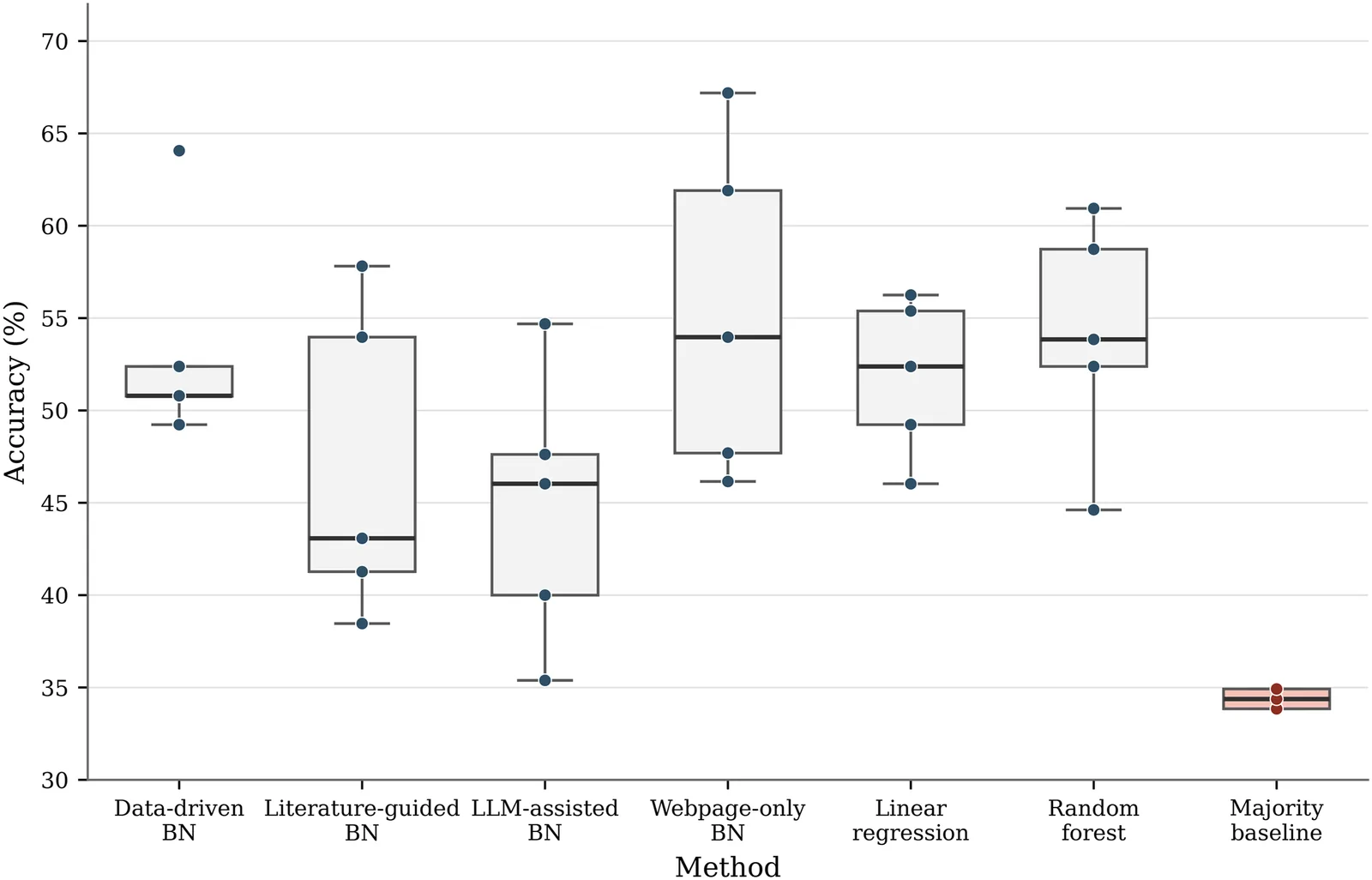
Fold-level accuracy for the six evaluated models and the fold-specific majority-class baseline in stratified five-fold cross-validation of the weekly dataset. Points denote held-out folds; boxes summarize the five fold accuracies.

For the three primary BNs, balanced accuracy was 0.529, 0.465, and 0.447, and macro-F1 was 0.515, 0.457, and 0.450, for the data-driven, literature-guided, and LLM-assisted models, respectively. Complete class-aware results are reported in S3 and S4 Tables, and confusion matrices are reported in S5 Table.

The weekly target comprised 110 Low, 103 Medium, and 107 High observations, and every evaluated model predicted all three classes. The predicted Low/Medium/High shares were 45.3%/23.1%/31.6% for the data-driven BN, 47.5%/24.7%/27.8% for the literature-guided BN, and 29.7%/41.6%/28.8% for the LLM-assisted BN; distributions for the other models are reported in S3 Table.

## Discussion

This study evaluated the predictive performance of Bayesian networks developed from the literature, LLM, and data for predicting angler pressure, measured as the number of boats observed in aerial surveys. Each BN exceeded the fold-specific majority baseline, and no pairwise difference among the six evaluated models remained significant after Holm adjustment. The modest macro-F1 and kappa values nevertheless indicate uneven recovery of the three boat-count categories. These results suggest that a recorded LLM-assisted structure can be evaluated as one candidate ecological model, but they do not establish equal effectiveness among construction strategies.

Webpage views were directly linked to the target variable in all three BN structures. This finding, together with the positive association between webpage views and aerially observed boat counts, is consistent with [12], who showed that webpage views contain useful information for predicting angler pressure. A plausible interpretation is that lake-specific webpage views reflect aggregate information-seeking or trip-planning behaviour: anglers choose whether and where to fish based on expected benefits, travel and time costs, site attributes, weather, and available information [14,15,64]. Lake webpages provide some of this information, including location, access points, target species, and other site characteristics [16,17]. Accordingly, viewing a lake webpage can be seen as part of the angler’s cost-benefit assessment: it lowers information-search costs and helps anglers update their knowledge of the expected utility of a lake before deciding whether to visit. This interpretation is also consistent with broader evidence that digital traces can help predict recreation and tourism activity [65,66]. The supplementary analyses support this interpretation only in a qualified sense: site-specific Wikimedia pageviews for national parks were positively associated with official monthly visitation, whereas broad national fishing-topic pageviews were weakly associated with NOAA recreational fishing effort. Thus, webpage views appear most defensible as local, site-specific predictive signals, not as general measures of recreational fishing effort.

Webpage views should therefore be interpreted as predictive indicators, not as causes or direct measures of realized fishing activity. A webpage view does not necessarily correspond to a fishing trip, and the observed association may also reflect lake popularity, repeated browsing, aspirational interest, stocking- or weather-related information seeking, or use by non-anglers. The 44.7–55.3% predictive accuracies observed here indicate that webpage views and other digital variables are useful but incomplete predictors of angler pressure. They are not accurate enough to replace aerial surveys, creel surveys, or other direct effort-estimation programs, and they should not be used on their own for harvest regulation, enforcement, or high-stakes management decisions. Their practical value is instead as a low-cost screening signal that may help identify lakes or periods where higher angler pressure is plausible and where follow-up monitoring would be useful.

The moderate accuracy should be interpreted in light of sparse and uneven platform coverage across lake-week combinations. The daily app variables and webpage views contained many zeros, which motivated weekly aggregation. These zeros may indicate true absence of reported activity, limited platform use, incomplete reporting, or low digital engagement for a given waterbody and date. The aerial-survey response variable also represents sampled open-water fishing-boat counts for the surveyed lakes and dates, not total angler effort across all lakes, seasons, or fishing modes. Accordingly, the models should be interpreted as testing whether available digital traces contain predictive information for the matched lake-week observations, not as estimating total fishing effort or representing the full angling population. Future work using denser citizen-sourced records and explicit measures of platform coverage could improve predictive accuracy and management relevance [67].

This approach should complement, not replace, current fisheries-monitoring practices. Creel/ intercept surveys, household or license-frame surveys, for-hire reporting, and aerial counts provide richer design-based estimates, but require substantial field effort and may be spatially or temporally coarse [68,69]. Mobile-device geolocation data may predict effort more directly when available, representative, ethically usable, and classifiable as fishing activity [70]. The BN approach is useful mainly as a transparent, low-cost framework for combining heterogeneous indicators where such mobility data are unavailable, restricted, or difficult to classify.

Bayesian networks were selected because they combine probabilistic prediction with an interpretable representation of dependence and have supported environmental prediction and decision support across eutrophication, native-fish risk, endangered-species habitat, cyanobacterial blooms, stream water quality, and fire-associated fauna distributions [71–76]. Related graphical-model work has produced useful spatial predictions under extreme ecological data scarcity [77]. Consistent with recommendations to compare expert- or literature-informed networks against data-driven alternatives and document expert intervention, we included automatic structure learning as a transparent comparator [78]; a learned structure can also be extended when a new variable is introduced rather than relearned from scratch [79].

The framework may be adaptable beyond Ontario open-water freshwater angling, but only with local validation. Similar BN models could be applied to other fisheries, beachgoing, hunting, or winter ice fishing if the response variable and predictors are replaced with activity-specific effort measures, such as shore or boat trips, beach counts, licence or harvest records, parking/access data, ice conditions, mobile-device indicators, or counts from aerial, drone, or satellite imagery. Saltwater applications would need to distinguish fishing modes, while winter ice-fishing models would require ice-specific effort measures rather than boat counts [8,70,80–82]. In all cases, citizen-sourced and web-based predictors should be treated as complementary screening tools, not replacements for statistically designed monitoring.

More broadly, this study adds to evidence that large language models can help formulate model structures, features, or hypotheses for empirical testing. LLM-assisted approaches have been used for few-shot tabular prediction [83], automated feature engineering [84], and applied forecasting workflows [85]. In ecology, recent work has begun to use language-derived representations for species-distribution modeling and to evaluate whether LLMs encode ecological knowledge useful for tasks such as species-presence prediction and range mapping [86]. Our contribution is to extend this line of research to recreational fisheries. This supports the cautious use of LLMs as model-structuring tools, provided that ecological applications remain grounded in domain expertise, transparent validation, and local data.

## Supporting information

S1 Fig An example of a Bayesian network. A Bayesian network illustrating conditional dependencies among random variables *A*,*B*,*C*,*D*,*E* that are discretized into three levels.
(PDF)

S2 Fig ChatGPT’s response to the provided prompt. ChatGPT’s proposed Bayesian network illustrating the relationships among the variables in response to the given prompt. The archived response contains an “is weekend” node because this variable belonged to the original daily feature set used to construct all three networks. Once the analysis was restricted to weekly aggregates, a single weekend indicator no longer had a meaningful definition and was therefore removed from every evaluated network.
(PDF)

S3 Fig Data-driven CPDAGs learned from the fold-1 and fold-2 weekly training partitions. Green nodes represent meteorological variables, yellow nodes represent webpage- and app-derived variables, and the coral node represents the aerial-survey boat-count target.
(PDF)

S4 Fig Data-driven CPDAGs learned from the fold-3 and fold-4 weekly training partitions. Node colors follow S3 Fig.
(PDF)

S1 TableAn example of a conditional probability distribution (CPD) for P(E∣B,C), showing the probabilities of different levels of *E* (Low, Medium, High) given combinations of *B* and *C.*Each row represents a specific condition of *B* and *C*, with corresponding probabilities for the levels of *E* in each column.(PDF)

S2 Table Distribution of key discretized weekly Ontario variables. Values are counts, with percentages in parentheses.
(PDF)

S3 Table Pooled out-of-fold weekly performance and predicted-class proportions. AUC is the Hand–Till multiclass AUC.
(PDF)

S4 Table Class-specific weekly precision and recall from pooled out-of-fold predictions. (PDF)

S5 Table Weekly pooled out-of-fold confusion matrices. Rows are actual classes and columns are predicted classes.
(PDF)

S6 Table Fold-level weekly accuracy. The final row gives the unweighted mean (SD) across the five held-out folds.
(PDF)

S7 Table Sensitivity of weekly BN classifications to CPT estimation. Unsupported rows are posterior vectors replaced because unsmoothed maximum-likelihood estimation supplied no supported probability.
(PDF)

S8 Table National Park Service contextual recreation-analogue dataset. Monthly NPS recreation visits with statistic code TRV were paired with monthly Wikimedia pageviews for the corresponding English Wikipedia article; these are not the Angler’s Atlas pageviews used in the Ontario model.
(PDF)
