# Supplementary material for: Comparing literature-guided, LLM-assisted, and data-driven models for predicting angler pressure
Source: PLoS One. 2026 Oct 9;21(10):e0357450. doi: 10.1371/journal.pone.0357450 (PMC13652327; doi:10.1371/journal.pone.0357450)
Supplement: Supplementary file 1 [file pone.0357450.s001.pdf]

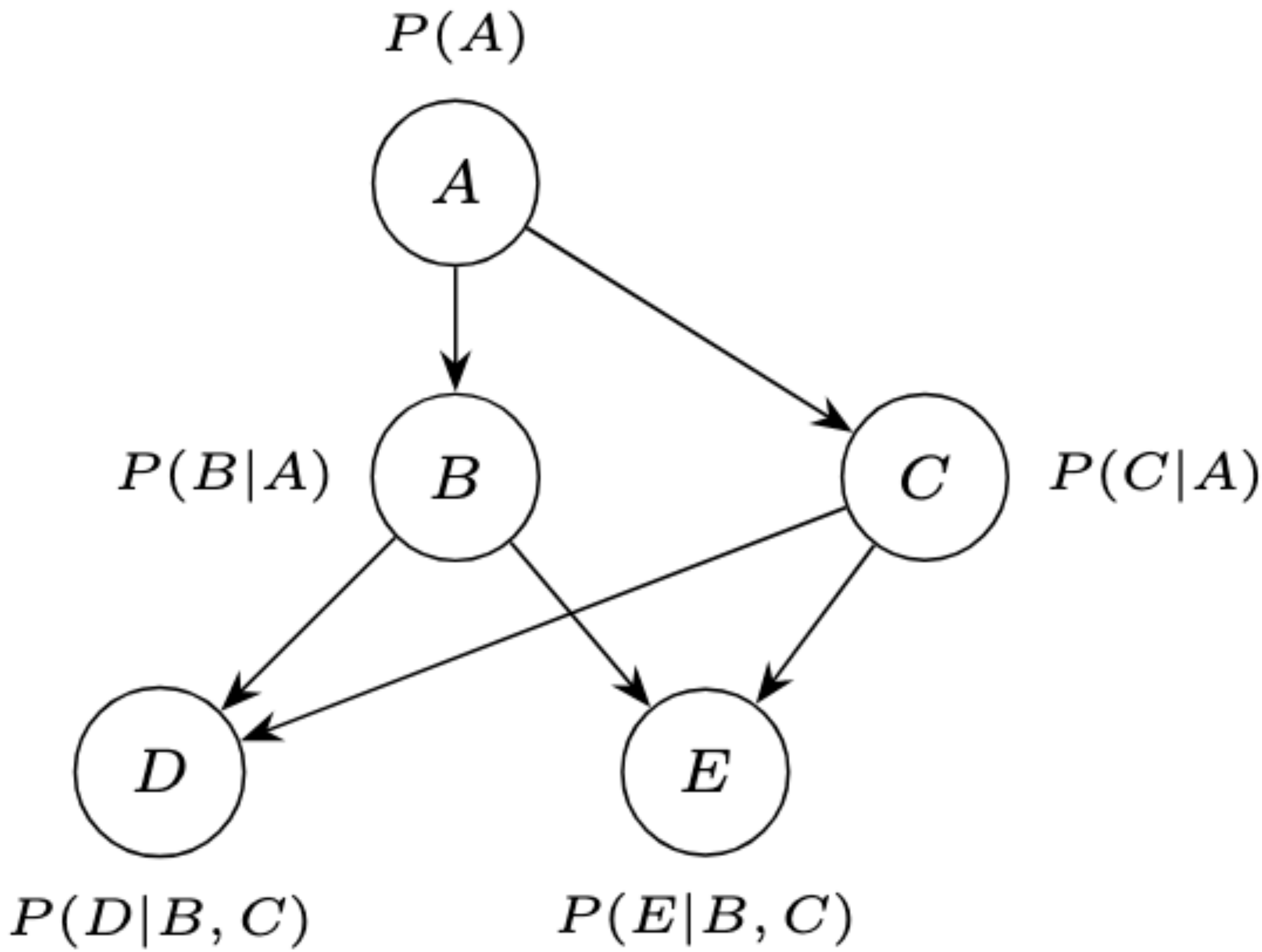

P(A)
A
P(B|A)
B
C
P(C|A)
D
E
P(D|B, C)
P(E|B, C)
