# Supplementary material for: Comparing literature-guided, LLM-assisted, and data-driven models for predicting angler pressure
Source: PLoS One. 2026 Oct 9;21(10):e0357450. doi: 10.1371/journal.pone.0357450 (PMC13652327; doi:10.1371/journal.pone.0357450)
Supplement: Supplementary file 2 [file pone.0357450.s002.pdf]

Bayesian Network Structure

1. **Weather Relationships**:
   - `relative_humidity` is influenced by `total_precipitation`, `air_temperature`, and `wind_speed`.
   - `air_temperature` is influenced by `degree_days` and `solar_radiation`.
   - `wind_speed` might influence `total_precipitation`.
2. **Fishing Activity Influences**:
   - `number_of_boats` is influenced by `is_weekend` and `web_page_views_last_seven_days`.
   - `app_catch_rate` is influenced by `number_of_boats`, `app_hours_out`, `air_temperature`, `solar_radiation`, `wind_speed`, and `relative_humidity`.
   - `app_hours_out` is influenced by `air_temperature`, `solar_radiation`, `wind_speed`, `relative_humidity`, and `is_weekend`.
   - `app_total_trips` is influenced by `is_weekend`, `web_page_views_last_seven_days`, and `air_temperature`.
   - `web_page_views_last_seven_days` can be influenced by `air_temperature`, `degree_days`, and `is_weekend`.

ChatGPT Auto

To visualize this, you can use a directed acyclic graph (DAG) where arrows indicate the direction of influence:

plaintext

Copy code

```
degree_days → air_temperature → relative_humidity
                                    ↓                 ↘  app_hours_out ↘  app_catch_rate
web_page_views_last_seven_days → number_of_boats      ↗                 ↗
                                            ↓               ↓   ↘    ↘
                                              app_total_trips   ↓     ↘
                                              ↓              ↓   ↓    ↘
                                        is_weekend → app_hours_out → app_catch_rate
                                              ↗             ↓           ↘
                                            number_of_boats →    app_catch_rate
                                              ↗               ↗
                                              ↗               ↓
                                            app_total_trips  ↓
                                                             ↓
                                           web_page_views_last_seven_days
```
