# Supplementary material for: Comparing literature-guided, LLM-assisted, and data-driven models for predicting angler pressure
Source: PLoS One. 2026 Oct 9;21(10):e0357450. doi: 10.1371/journal.pone.0357450 (PMC13652327; doi:10.1371/journal.pone.0357450)
Supplement: Supplementary file 5 [file pone.0357450.s005.pdf]

	E=Low	E=Medium	E=High
B=Low, C=Low	0.7	0.2	0.1
B=Low, C=Medium	0.3	0.6	0.1
B=Low, C=High	0.7	0.1	0.2
B=Medium, C=Low	0.3	0.4	0.3
B=Medium, C=Medium	0.1	0.2	0.7
B=Medium, C=High	0.2	0.2	0.6
B=High, C=Low	0.1	0.6	0.3
B=High, C=Medium	0.2	0.1	0.7
B=High, C=High	0.4	0.4	0.2
