# Supplementary material for: Comparing literature-guided, LLM-assisted, and data-driven models for predicting angler pressure
Source: PLoS One. 2026 Oct 9;21(10):e0357450. doi: 10.1371/journal.pone.0357450 (PMC13652327; doi:10.1371/journal.pone.0357450)
Supplement: Supplementary file 6 [file pone.0357450.s006.pdf]

Dataset	Variable	N	Low	Medium	High
Weekly	Number of boats	320	110 (34.4)	103 (32.2)	107 (33.4)
Weekly	Webpage views	320	123 (38.4)	102 (31.9)	95 (29.7)
Weekly	App number of trips	320	197 (61.6)	36 (11.3)	87 (27.2)
Weekly	App fishing duration	320	110 (34.4)	104 (32.5)	106 (33.1)
Weekly	App catch rate	320	116 (36.3)	102 (31.9)	102 (31.9)
