# Supplementary material for: Comparing literature-guided, LLM-assisted, and data-driven models for predicting angler pressure
Source: PLoS One. 2026 Oct 9;21(10):e0357450. doi: 10.1371/journal.pone.0357450 (PMC13652327; doi:10.1371/journal.pone.0357450)
Supplement: Supplementary file 7 [file pone.0357450.s007.pdf]

Model	Accuracy	Balanced accuracy	Macro-F1	Kappa	AUC	Pred. Low	Pred. Medium	Pred. High
Data-driven BN	0.534	0.529	0.515	0.299	0.719	0.453	0.231	0.316
Literature-guided BN	0.469	0.465	0.457	0.200	0.663	0.475	0.247	0.278
LLM-assisted BN	0.447	0.447	0.450	0.172	0.568	0.297	0.416	0.288
Webpage-only BN	0.553	0.550	0.550	0.329	0.685	0.384	0.319	0.297
Linear regression	0.519	0.521	0.528	0.282	0.739	0.234	0.528	0.238
Random forest	0.541	0.536	0.528	0.309	0.735	0.400	0.259	0.341
Majority baseline	0.344	0.333	0.171	0.000	–	1.000	0.000	0.000
