# Supplementary material for: Comparing literature-guided, LLM-assisted, and data-driven models for predicting angler pressure
Source: PLoS One. 2026 Oct 9;21(10):e0357450. doi: 10.1371/journal.pone.0357450 (PMC13652327; doi:10.1371/journal.pone.0357450)
Supplement: Supplementary file 8 [file pone.0357450.s008.pdf]

Model	Class	Support	Precision	Recall
Data-driven BN	Low	110	0.538	0.709
	Medium	103	0.324	0.233
	High	107	0.683	0.645
Literature-guided BN	Low	110	0.467	0.645
	Medium	103	0.367	0.282
	High	107	0.562	0.467
LLM-assisted BN	Low	110	0.526	0.455
	Medium	103	0.346	0.447
	High	107	0.511	0.439
Webpage-only BN	Low	110	0.593	0.664
	Medium	103	0.382	0.379
	High	107	0.684	0.607
Linear regression	Low	110	0.547	0.373
	Medium	103	0.373	0.612
	High	107	0.816	0.579
Random forest	Low	110	0.563	0.655
	Medium	103	0.361	0.291
	High	107	0.651	0.664
