# Supplementary material for: Comparing literature-guided, LLM-assisted, and data-driven models for predicting angler pressure
Source: PLoS One. 2026 Oct 9;21(10):e0357450. doi: 10.1371/journal.pone.0357450 (PMC13652327; doi:10.1371/journal.pone.0357450)
Supplement: Supplementary file 9 [file pone.0357450.s009.pdf]

Model	Actual class	Pred. Low	Pred. Medium	Pred. High
Data-driven BN	Low	78	25	7
	Medium	54	24	25
	High	13	25	69
Literature-guided BN	Low	71	24	15
	Medium	50	29	24
	High	31	26	50
LLM-assisted BN	Low	50	42	18
	Medium	30	46	27
	High	15	45	47
Webpage-only BN	Low	73	29	8
	Medium	42	39	22
	High	8	34	65
Linear regression	Low	41	67	2
	Medium	28	63	12
	High	6	39	62
Random forest	Low	72	29	9
	Medium	44	30	29
	High	12	24	71
