# Supplementary material for: Comparing literature-guided, LLM-assisted, and data-driven models for predicting angler pressure
Source: PLoS One. 2026 Oct 9;21(10):e0357450. doi: 10.1371/journal.pone.0357450 (PMC13652327; doi:10.1371/journal.pone.0357450)
Supplement: Supplementary file 10 [file pone.0357450.s010.pdf]

Table S6: Fold-level weekly accuracy. The final row gives the unweighted mean (SD) across the five held-out folds.

Fold	Data-driven BN	Literature-guided BN	LLM-assisted BN	Webpage-only BN	Linear regression	Random forest	Majority baseline
1	0.492	0.431	0.354	0.477	0.492	0.446	0.338
2	0.508	0.385	0.400	0.462	0.554	0.538	0.338
3	0.641	0.578	0.547	0.672	0.563	0.609	0.344
4	0.508	0.413	0.460	0.540	0.460	0.524	0.349
5	0.524	0.540	0.476	0.619	0.524	0.587	0.349
Mean (SD)	0.534 (0.060)	0.469 (0.085)	0.447 (0.074)	0.554 (0.091)	0.519 (0.043)	0.541 (0.063)	0.344 (0.005)
