# Supplementary material for: Comparing literature-guided, LLM-assisted, and data-driven models for predicting angler pressure
Source: PLoS One. 2026 Oct 9;21(10):e0357450. doi: 10.1371/journal.pone.0357450 (PMC13652327; doi:10.1371/journal.pone.0357450)
Supplement: Supplementary file 11 [file pone.0357450.s011.pdf]

Estimator	Model	Accuracy	Unsupported rows
Dirichlet, ISS = 1	Data-driven BN	0.534	0
	Literature-guided BN	0.469	0
	LLM-assisted BN	0.447	0
Dirichlet, ISS = 5	Data-driven BN	0.534	0
	Literature-guided BN	0.469	0
	LLM-assisted BN	0.447	0
Dirichlet, ISS = 10	Data-driven BN	0.534	0
	Literature-guided BN	0.469	0
	LLM-assisted BN	0.447	0
Maximum likelihood	Data-driven BN	0.534	0
	Literature-guided BN	0.469	0
	LLM-assisted BN	0.406	59
