# Supplementary material for: Comparing literature-guided, LLM-assisted, and data-driven models for predicting angler pressure
Source: PLoS One. 2026 Oct 9;21(10):e0357450. doi: 10.1371/journal.pone.0357450 (PMC13652327; doi:10.1371/journal.pone.0357450)
Supplement: Supplementary file 12 [file pone.0357450.s012.pdf]

| **Park** | **NPS code** | **Wikipedia article** |
|---|---|---|
| Acadia | ACAD | `Acadia_National_Park` |
| Arches | ARCH | `Arches_National_Park` |
| Bryce Canyon | BRCA | `Bryce_Canyon_National_Park` |
| Canyonlands | CANY | `Canyonlands_National_Park` |
| Capitol Reef | CARE | `Capitol_Reef_National_Park` |
| Crater Lake | CRLA | `Crater_Lake_National_Park` |
| Cuyahoga Valley | CUVA | `Cuyahoga_Valley_National_Park` |
| Death Valley | DEVA | `Death_Valley_National_Park` |
| Everglades | EVER | `Everglades_National_Park` |
| Glacier | GLAC | `Glacier_National_Park_(U.S.)` |
| Grand Canyon | GRCA | `Grand_Canyon_National_Park` |
| Grand Teton | GRTE | `Grand_Teton_National_Park` |
| Great Smoky Mountains | GRSM | `Great_Smoky_Mountains_National_Park` |
| Joshua Tree | JOTR | `Joshua_Tree_National_Park` |
| Mount Rainier | MORA | `Mount_Rainier_National_Park` |
| Olympic | OLYM | `Olympic_National_Park` |
| Rocky Mountain | ROMO | `Rocky_Mountain_National_Park` |
| Yellowstone | YELL | `Yellowstone_National_Park` |
| Yosemite | YOSE | `Yosemite_National_Park` |
